# The Rise and Fall of Energy Democracy: 5 Cases of Collaborative Governance in Energy Systems

**DOI:** 10.1007/s00267-022-01687-8

**Published:** 2022-07-29

**Authors:** Olivier Berthod, Thomas Blanchet, Henner Busch, Conrad Kunze, Colin Nolden, Michelle Wenderlich

**Affiliations:** 1 ICN Business School & CEREFIGE, Université de Lorraine, Nancy, France; 2 Nexus Institute for Cooperation Management and Interdisciplinary Research, and Technical University of Berlin, Berlin, Germany; 3 Lund University Centre for Sustainability Studies, Lund, Sweden; 4 Comparative Politics/Environmental Policy Research Centre (FFU) at Freie Universitaet, Berlin, Germany; 5 Centre for Research into Energy Demand Solutions, Environmental Change Institute, University of Oxford, Oxford, UK; 6 UK Energy Research Centre, Law School, University of Bristol, Bristol, UK; 7 Independent Scholar, PhD, Clark University Graduate School of Geography & Rochester for Energy Democracy at MetroJustice, Worcester, MA USA

## Abstract

A wide range of actors are seeking to democratize energy systems. In the collaborative governance process of energy system transitions to net zero, however, many energy democracy concepts are watered down or abandoned entirely. Using five renewable energy case studies, we first explore the diversity of energy democratizing system challengers and bottom-up actors. Secondly, we analyze the role of conflict and challenges arising from the subsequent collaborative governance process and identify what appear to be blind spots in the CG literature. Our case studies on Berlin (GER), Jena (GER), Kalmar (SWE), Minneapolis (US) and Southeast England (UK) include different types of policy processes and actors. They suggest that actors championing energy democracy principles play an important role in opening participation in the early stages of collaborative energy transition governance. As collaborative governance progresses, participation tends to be increasingly restricted. We conclude that collaborative processes by themselves are insufficient in maintaining energy democracy principles in the energy transition. These require institutional embedding of participative facilitation and consensus building. The Kalmar case study as our only successful example of energy democracy suggests that a more intermediated and service-oriented approach to energy provision can create a business case for democratizing energy provision through collaborative governance.

## Introduction

A transition from fossil fuels towards renewable energy systems (RES) is essential for a low or zero carbon economy. The recognition of the social and political nature of this transition and especially its grassroots actors is commonly referred to as energy democracy (ED). It conveys a promise of greater participation and pluralistic control over power production and distribution (Becker and Naumann 2017; Szulecki 2018). ED projects hold the potential to establish and strengthen local decision-making processes and democratic institutions (Islar and Busch 2016; Szulecki and Overland 2020; Wahlund and Palm 2022). They instill a notion of ecological citizenship (Kenis 2015) as they are usually related to a range of public, non-profit and community-based efforts (Kunze and Becker 2014). They sometimes enable RES deployment where they are prevented by a lack of scale economies and high transaction costs (Nolden et al. 2020). Many ED initiatives also try to establish decentralized and local forms of ownership (Walker and Devine-Wright 2008; van Veelen 2018). However, they often have to compete against political, economic and social interests of incumbent actors (Rogers et al. 2008; Blanchet 2015; Becker et al. 2016). Their success often depends on a renegotiation of the roles of all participants involved to potentially shift the embedded goals of the energy systems at stake.

Public administration and governance scholars agree that collaborative structures are increasingly the policy tool of choice to deliver public services and administrate programs (Milward and Provan 2000; Moynihan 2008). Collaborative governance (CG) refers to a self-referential group of purposefully interconnected state and non-state organizations engaged in collective decision-making processes that are formal, consensus-oriented, and deliberative (Ansell and Gash 2008). Although, most treatments of CG focus on groups of organizations that have agreed upon collaborating in the first place or are bound to do so by some level of interdependence in their action. Therefore, the context of ED offers an interesting contrast insofar as societal actors often organize themselves to participate in policy making about energy. Relying on both frameworks, this article aims to assess how CG mechanism influence the ED principles.

In this paper, we explore the following research questions: *How do societal actors attempt to introduce ED concepts into local energy systems, and how do CG processes affect their outcome?* We selected five case studies that involve a range of non-traditional energy actors based on their varying level of involvement with ED and nuanced CG processes. Two cases offer insights into early ED efforts at the stage of issue campaigning for more CG that led to new formal arrangements with regards to more collaboration in the energy sector. Two cases offer insights into ED efforts that were halted before delivering on their goals as a result of the CG process. The last case was a success regarding its ambitions. We thereby covered a wide breadth of policy development stages, from cases on agenda setting to cases on service implementation and operation. The covered time periods span up to 10 years.

Our results emphasize the limited ability of non-governmental and non-profit actors to navigate tensions and democratize local energy policies against strong incumbent interests. We demonstrate how grassroots movements often act conflictive, which can be beneficial at certain phases of the CG process. We also identify power asymmetry characteristics in existing energy systems that result from the prioritization of shareholder profit, rigidities of public as well as private actors in decision-making, and path dependency (Arthur 1989; Kuzemko et al. 2016). These factors limit the potential of ED approaches maintaining their momentum in the CG process of energy system change.

## Theoretical Background

### Energy Democracy

The concept of ‘energy democracy’ (ED) first emerged in the discourses of social movements and civil society. They saw the decarbonization of the energy system as an opportunity to change the means of control over energy production and distribution (Strachan et al. 2015) and, more generally, to achieve deeper economic and socio-political transformations (Burke and Stephens 2017). In recent years, this concept has been subject of increasing interest from the scientific community (van Veelen and van der Horst 2018; Szulecki and Overland 2020; Wahlund and Palm 2022). ED promotes the opening up of the energy sector to new actors and, in particular, to ‘prosumers’ (simultaneously producers and consumers), energy cooperatives, social enterprises or companies under municipal control (Szulecki 2018).

Szulecki (2018) distinguishes between ED as a ‘quasi-utopian idealization’ and a more concrete process influencing the energy system and its actors. Becker and Naumann (2017) furthermore identify essential elements important in different struggles: decentralization of energy systems, engagement of citizens in the decision-making process, collective (public and cooperative) forms of ownership, substantial economic benefits associated with energy activity, and self-determination and alternatives to extractivist development. Others characterize ED by its objectives, such as correcting structural inequalities linked to energy policies (Hess 2018), and resolving situations of energy insecurity and accessibility for the most marginalized populations (Teron and Ekoh 2018).

There has been a surge of ED in the last decade (Kunze and Becker 2014; Vansintjan 2015; Fairchild and Weinrub 2017; Szulecki and Overland 2020). The projects allow citizens to be politically, socially and financially involved in the production, distribution and use of renewable energies. ED as a process can thus be understood as a tendency towards (re-)embedding the economy in society, with regards to the energy sector (in the sense of Polanyi, 1944). Literature from Europe and northern America attributes higher popular acceptance of energy transitions to ED projects as they support the financial and political involvement of local populations (Vansintjan 2015; Bauwens et al. 2016; Becker and Naumann 2017; Brummer 2018; Szulecki 2018; Mundaca et al. 2018; Klagge and Meister 2018; Busch et al. 2021). The situation in the rest of the world, especially in nations with weak democratic institutions, has been often found to be very different if not detrimental. RES in the global south are often related to human rights abuses and corruption, similar to fossil energies, and there are very few processes that would qualify as ED (Avila-Calero 2018; Dunlap 2021).

ED is often equated with community energy (van Veelen 2018). Community energy involves the setting up of a dedicated entity, such as a charity or non-profit association, a limited company, or a citizens’ cooperative (Creamer et al. 2018). Social entrepreneurial tendencies as a result of market pressures and tight margins can however result in practical limits to their openness and participatory nature (Nolden et al. 2020). Less well researched but equally important in the context of ED are collaborative policy processes involving a variety of other participants involved in agenda-setting around democratization and decentralization such as civil society organizations, associations of users, political parties, city councils and administrations, incumbent as well as interested utilities, among other examples (see e.g. Becker and Naumann 2017). At the same time, a growing literature examines the dynamics and challenges of remunicipalizing energy and/or democratizing public energy systems (Blanchet 2015; Becker et al. 2016; Angel 2017, 2021; Brinker and Satchwell 2020).

More than any of these characteristics, however, the social movement roots of ED stress the need to change power relations and shift energy system outcomes towards environmental, social, racial, and economic justice and equality (see e.g. Angel 2016; Fairchild and Weinrub 2017). The democracy element of ED can have multiple meanings: forms of collective ownership or control; processes of internal participatory democracy; democratic access and equality, where energy is decommodified and not serving profit motives; just distributions of who profits and who suffers (Wenderlich 2021). Energy democracy outlines an alternative development path for the energy system, one that can serve to address root causes of uneven and extractive development models at the heart of the climate crisis. It is this emphasis on socio-ecological transformation that especially challenges collaborative governance frameworks (adapted from Heldeweg and Saintier 2020).

ED projects and processes favor accountability and participation. The more they do so, however, the more they must navigate important tensions, as they run on rationales that challenge the logics of homogenous commercial or state enterprises in order to support the assemblage of heterogeneous actors (Rogers et al. 2008; Bauwens and Devine-Wright 2018) and cope with institutional complexity (Bauwens et al. 2022). Much more complex to steer than single organizations, such collaborative structures are highly dependent on beneficial local contexts and thrive in symbiosis with the capacity for local participants to reach consensus, as well as overarching political, organizational and legal frameworks, which either support or hinder the democratization of energy systems.

### Collaborative Governance Regimes

As mentioned above, changes in governance such as those that democratize energy or transition energy systems towards zero carbon are an illustration of CG dynamics more generally. The literature on CG relies on a variety of academic traditions, including research streams on collective action and common resource pools (Ostrom 1990), public network management (O’Toole 1997), or deliberative and participatory democracy (Bingham 2011). The frameworks that have emerged around the notion of CG thus cover a wealth of phenomena, such as the research on policy networks (e.g. Klijn 1996), inclusive management (e.g. Quick and Feldman 2014), purpose- and goal-oriented networks (e.g. Provan et al. 2007; Nowell and Kenis 2019), or multi-stakeholder and sectoral partnerships (e.g. Herranz 2008).

According to Ansell and Gash (2008), CG is concerned with collaborations among formally independent organizations across sectors. These collaborations are consensus-oriented and produce collective outputs in the form of decisions, plans, services, that are attributed to the collaborative and not to one participant only. Against this background, Emerson et al. (2012, p.2) insist on the fact that CG includes “processes and structures of public policy decision making and management that engage people constructively across the boundaries of public agencies, levels of government, and/or the public, private and civic spheres in order to carry out a public purpose that could not otherwise be accomplished.” This view further echoes the wide spectrum of policy processes aforementioned, from participatory governance and civic engagement to public network management, and many other instances of cross-sector partnerships.

A major distinction of the CG framework by Ansell and Gash (2008) is its interest with the inner structure, organization and functioning of the collaboratives. Specifically, Ansell and Gash (2008) conducted an analysis of 137 cases that were using a variety of models and policy processes (from deliberative to highly structured instances of collaboration) with an interest in starting conditions as well as in other inner variables that influence a collaborative’s success. Effective collaborative governance requires situated combinations of four specific dimensions. Adequate starting conditions include the history of conflict and cooperation, power, resources and knowledge asymmetry, incentives and interdependence between actors. Secondly, institutional design (e.g. explicit rules, transparency of decision-making) and some form of facilitative leadership (e.g. ability to convene actors or mediate in conflicts) impact the collaborative process. And the collaborative process itself builds on face-to-face dialogue, commitment to process, trust building, shared understanding, and achieving intermediate results.

Amid this interest in the internal dynamics of collaboration that are conducive of effective CG regimes, research from a variety of policy domains have shown that tensions, such as the ones we discussed regarding ED, are inherent characteristics of collaborative governance (Ansell and Gash 2008; Provan and Kenis 2008; Saz-Carranza and Ospina 2011; Berthod and Segato 2019). Focusing on the tensions amounts to a conception of CG as a set of co-existing, key contradictory forces that need to be reconciled in practice for ED to succeed. Tensions in CG can concern a variety of issues, such as the pull between collective and organizational resources, interdependence versus autonomy, integration versus fragmentation, or inclusivity versus efficiency, to name but a few (see Berthod and Segato 2019 for a review). A failure to attend and resolve such tensions can doom collaborative efforts at any maturity stage (Human and Provan 2000; Cornforth et al. 2015). This perspective offers a particularly dynamic view on CG efforts and their development, shedding light on their fragility as they evolve.

As Ulibarri and colleagues (2020) highlight, CG arrangements often experience turbulent beginnings. These turbulent early phases lead to a working mode that promotes stability. In the particular case of ED, however, extant frameworks point to the role of bottom-up actors challenging incumbents and attempting to change the embedded values of the energy system, along with its processes, in creating conflictual relations. At the end of the road, the energy system is notable for the intransience and difficulty of shifting actors locked into past configurations (Arthur 1989; Kuzemko et al. 2016). It is these issues of the importance of the specificity of actors (grassroots or public) attempting specific changes (non-profit orientation and opening of process and decision-making along with decentralization of physical infrastructure) up against a system with strong incumbency dynamics, that challenges CG in ED efforts. CG approaches are ambivalent about the type of actors involved and the ends they are seeking (Herranz 2008), while ED proposes democracy as a mechanism for paradigm change and shifting power relations. Therefore, we explore efforts to introduce ED concepts into local energy systems and the tensions that arise from ambitions to increase societal participation in the CG of energy system change.

## Methods

Our methodological design uses the CG framework by Ansell and Gash (2008) as a set of conceptual devices to explore cases of ED because it allows us to capture context and starting conditions as well as the internal functioning of CG arrangements. Grounded in empirical research, this framework has been thoroughly empirically tested since its publication (Geerling and Smits 2016; Douglas et al. 2020b) and became the object of a recent database that offers a platform and tools for case comparisons (Ansell and Gash 2008, p. 544, the top-down approach, and Stirling 2019, the bottom-up approach).

We rely on the material from 5 case studies. These 5 cases have been selected among the research projects the various members of the research team have been involved in. We identified the most relevant in the context of collaborative governance. The cases offer variations in terms of policy implementation. Upstream in the policy process, two cases, one from Berlin, Germany, and one from Minnesota, US, offer insights into efforts in civic engagement and agenda-setting about democratic control over energy systems, and later attempts at grassroots policy implementation. Midstream in the policy process, a case from Jena, Germany, gives insights in institutionalized changes in the governance structure of the communal energy supply. And further downstream, two more cases, one from the UK and one from Sweden, offer insights into ED projects that imply not only institutionalized changes in governance but also changes in energy production and supply, as well as governance and benefit arrangements. In Table 1, we briefly outline the main developments and data sources for each of the five cases.

**Table 1:** Overview of cases

	JENA (GER)	MINNEAPOLIS (US)	RIDING SUNBEAM (UK)	BERLIN (GER)	KALMAR (SWE)
The case	Acquisition of shares in the municipal energy company by a citizen’s cooperative	Minneapolis energy options campaign, an initiative to remunicipalize electricity services	Development of a prototype of solar power traction for trains to pave the way for community and commuter co-owned tracton power supply assets	Energietisch, a civil society initiative wrote a law proposal suggesting the creation of a publicly owned and direct democratically controlled grid operator and public energy supplier	Joint launch and operation of solar parks in Kalmar by Kalmar Energi and a consortium of businesses, citizens, and public entities
Original goal of initiators	Promote a decentralised, affordable, environmentally and climate-friendly energy supply based on 100% renewable energy sources	A public option and local control could accelerate the renewable transition and make it accessible to all, benefit the local economy, and lower prices	Research and development: can we provide power traction for trains using solar energy?	Act against energy poverty and reach 100% regional renewables, ability for universal participation of residents	Not explicit
Initiators	Key players in the local semi-private utilities and from the municipality	Local civil society groups with some facilitation from a city council member	10:10 Climate Action (now We Are Possible) and Community Energy South	A coalition of 56 civil society groups	Kalmar Energi (the local public energy company)
Participants	Citizen-owned cooperative, municipal energy company, city council	Local civil society groups, city council and the privately owned utilities, along with a resident’s advisory council	Public funding institutions, not-for-profit parent companies, local universitities, Network Rail	56 local civil society groups, local political parties and ministries, later the new public utility (with advisory council) and public grid operator	Municipality, the publicly-owned energy provider, the Kalmarsund Sol community energy association representing participating inhabitants, and local businesses
Outcome	The cooperative acquired 2% of the public utility; eventually, the cooperative decided to distribute dividends and was blocked by the council as it tried to acquire more shares. Investments in renewables stalled.	City council refused to put remunicipalization on the ballot; the Clean Energy Partnership (CEP) between the city and its utilities as an attempt at long-term collaborate governance emerged.	Collaboration until roll out. ‘First Light’ demonstrator on the Wessex Route at Aldershot in 2019 was the world’s first case of solar power directly powering commercial trains; route to market blocked by difficulties to scale	The draft was presented for vote in a referendum, missed the quorum by 20.000 vote. Eventually, electric grid municipalization and public utility emerged, but with limited democratic/social vision and market power.	Sweden’s biggest solar park as of 2016; Kalmar Energi has become the national expert for community energy projects
Data	4 interviews with 8 Interviewees, 41 documents, 1 observation	75 interviews, document analysis, 6 months+ participant observation	21 interviews, 15 documents, 8 observations, - surveys	45 interviews, document analysis, 6 months+ participant observation	5 interviews, 20+ documents

To explore our five cases, we relied on multiple approaches. In a first step, we reported our cases and related material systematically with the help of the standard form on collaborative governance developed by the Collaborative Governance Case Database project. The five cases were submitted for inclusion in the database. Recently launched (Douglas et al. 2020a), the database offers an opportunity to leverage the combined work of collaborative governance researchers around the globe. The case database is a common pool resource that shares high quality, previously published case studies. The main advantage of this tool for our purpose is that our case studies could be processed and analyzed using the many variables of interest from the model by Ansell and Gash (2008). The data in these case reports range from descriptions of the starting conditions of the collaboration to assessments of the performance of the collaboration as such. The data is captured both through quantitative scores on Likert Scales (e.g. “To what extent did the participants have more or less equal levels of resources to bring to the collaborative process?” (1 = Highly unequal, 5 = Highly equal)) and through open, long-form questions, such as: ‘describe the sense of interdependence between the actors’). Each case report is about 17 pages long. Each case author in our team filled in their respective form using the data material they had assembled on their case and related project reports and papers. The form further gives space for narratives on each dimension of the framework, which helped us to make sense of the assigned scores. The forms then circulated among all of us for feedback and questions.

We then explored this case material using the performance of the collaboration as a central variable of interest. In line with the procedures used by Douglas and colleagues (Douglas et al. 2020b), performance was delineated by the combination of items in the form: (1) effectiveness at reaching the project’s own goals (as goals were different between projects); (2) legitimacy (inferred from the support received from stakeholders); (3) support for future engagement. To orient our analyses, we then developed a table (see Table 2) for comparing our cases more systematically. We built this table using Douglas and colleagues’ translation of the Ansell and Gash model and filled the required variables with information lifted from our case forms. Most of the items in the form ask three Likert scores to cover the start, middle and end of the period observed. One of us took all the forms and assigned estimates (“-” for weak to very weak development, “+” for strong to very strong development, and “m” for moderate development) to represent the developing presence or absence of specific items in the cases over time. Specifically, a “-” was assigned to items that reported either a decrease or stagnation below 3 over time. Conversely, a “+” was assigned to items reporting either an increase or stagnation of 3 and more over time. The category “m” was assigned for scores converging to a 3 or whenever we faced a more nuanced development; for example, when items increased from start to middle, but decreased eventually. We then discussed the results collectively and altered single interpretations when needed.

**Table 2:** Cases comparison

	JENA (GER)	MINNEAPOLIS (US)	RIDING SUNBEAMS (UK)	BERLIN (GER)	KALMAR (SWE)
Theroetical sampling					
Stage in policy development	Governance change	Agenda-setting and governance change	Service implementation	Agenda-setting and governance change	Service implementation and operation
Performance	Weak	Weak	Moderate	Moderate	Good
Effectiveness	–	m	m	m	+
Legitimacy	m	–	+	m	+
Support for the future	–	m	m	+	+
Starting conditions					
Prehistory of cooperation or conflict (trust)	+	–	m	–	+
Balanced access to resources and knowledge	+	m	–	–	m
Incentives/interdependence	+	+	m	m	m
Institutional design					
Participatory inclusiveness	m	–	–	–	m
Clear ground rules	+	m	–	–	+
Application of said rules	+	m	–	m	+
Process transparency	+	m	–	m	+
Facilitative leadership					
Representing stakeholders	m	m	m	m	m
Mitigate conflicts	m	–	m	–	+
Maintaing procedural integrity	–	m	m	m	+
Collaborative process					
Face to face dialogue	+	+	+	m	+
Intermediate outcomes (small wins, plans, …)	m	m	+	m	+
Commitment to process	–	m	m	m	m
Shared understanding (mission, definitions, values)	–	–	m	–	+
Trust building	m	–	m	m	+

+: Strong to very strong development of characteristic

m: Moderate development of characteristic

–: Little to no development of characteristic

Table 2 helped us reduce the mass of information and details down to the cases’ quintessence in terms of development over time and outcomes. Referring to this table, we see that the Kalmar case corroborates predictions by the framework. Good starting conditions, as well as efforts in institutional design and facilitative leadership over time are conducive of a positive development in terms of collaborative process, which yields positive results for ED initiatives. The other four cases, however, offer more nuances. The UK and Berlin cases show that poor starting conditions and poor institutional designs can be compensated by moderate efforts in leadership, since this seems to have fed a moderate to good collaborative process, which yielded moderately good results, albeit to the detriment of some core ED principles. The Minneapolis case shows that a good collaborative process, however, will not necessarily emerge out of moderate efforts in institutional design and leadership. Specifically, poor developments in terms of shared understanding and the emergence of distrust seem to jeopardize efforts towards ED. Even more intriguing, the Jena case shows that good starting conditions and institutional design, as well as moderate leadership efforts are by no mean a guarantee for success. In this specific case, the collaborative process derailed all the same, which seems to have undermined performance.

The nuanced relation between process on the one hand, and design and leadership on the other in these first evaluations led us to inspect potential struggles at play to account for any lack of development using the qualitative answers in the forms. To do that, we used a definition by Provan and Kenis (2008), who propose to conceive of tensions in collaboratives as the existence of contradictory logics at network level; for example, when collaborators decide to focus on efficient decision-making in smaller groups, thereby reducing the beneficial influence of maximizing inclusiveness in the group. In this final phase of our analysis, we used an inductive approach as we skimmed through the open answers in the forms and identified the obstacles to the collaborative process. Thereby, we identified two dichotomous processes, which we will expose later in the discussion: *restricting* versus *opening*. In the next section, we present our findings in the form of five short narratives that provide focused, summarized insights into how these elements were played out in each case, thereby contributing to success or demise of the respective ED approaches in the context of CG processes.

## Case Observations

### Berlin, Germany

The Berlin case began with comparatively poor starting conditions. Energietisch, a coalition of over 50 groups, initiated collaboration as a means to remunicipalize energy in the city-state of Berlin. This idea, however, was at odds with the incumbent system and major political forces in government (Blanchet 2015). The group opened participation as it used plenary sessions and civic participation to write a referendum law proposing the creation of a publicly owned and democratically controlled grid operator and public energy supplier. The referendum law, among other things, set explicit goals against energy poverty, aimed to reach 100% regional renewables, asked for a board with seats for employees and elected members, as well as yearly assemblies and tools to petition the board. Social justice and democratic control were ends in themselves, as well as mechanisms through which a decentralized and ecological energy transition could be implemented and the main protagonists held accountable.

Due to its grassroots and conflicting origins, this movement never became part of a new, collaborative regime, in which it would contribute to develop ground rules and see these rules applied. In line with an orientation to openness, most of the internal organization of the movement was democratic. There was a steering committee, a few prominent campaign speakers and a paid campaigner, but decisions were made collectively through voting in open plenaries. There were working groups to help develop the social, ecological, and democratic elements, and lawyers to help draft the collective proposals into legal language. What is more, the movement successfully contributed to increase civic participation in energy policy and demonstrate its legitimacy in representing the people’s will. Once the proposed referendum law was developed, the coalition had to go through two rounds of signature collection – one collecting about 20,000 signatures, the second about 200,000. The structure of referendum law in Berlin is that initiatives can write their own laws to be voted on with binding implementation if they are approved, but 25% of eligible voters have to vote yes. Amid these developments towards inclusiveness, the city took action that restricted participation in the energy system. In a context where the government thought the referendum too far-reaching, the city postponed the date of the referendum vote, which was supposed to take place together with federal elections for practical reasons, to a different date a few months later, thereby provoking distrust regarding procedural integrity (Becker et al. 2016). Nevertheless, 600,000 Berliners came out to vote overwhelmingly in favour (82%), but missed the participation quorum by 0.9%, meaning that the government didn’t have to implement the result.

Although the coalition did not meet its goals of commoning, it did force a number of intermediate and, perhaps more surprisingly, delayed outcomes in the city. The overwhelming result of the referendum pressured the Berlin government towards taking on the issue of renewable energy (albeit at a diminished scale), while setting aside the social and democratic elements. Shortly before the referendum took place, the Berlin government created a public utility to sell renewable energy and compete with the incumbent firms; this utility, however, was supposed to produce renewable energy itself. In 2017, a new government lifted most of these limitations. The public utility is now a major actor for solar and energy retrofit projects for the public sector and for tenant energy subscriptions, although its customer numbers have grown slowly. Although this represents a very limited version of the original demands by Energietisch, the advisory council of the public utility contributes to offering a public forum and more transparency for face-to-face discussion on energy policy for a variety of stakeholders. There are on-going formal and informal talks between Energietisch actors and the by now two public utilities (in 2021 Berlin bought the grid back).

### Minneapolis, USA

The Minneapolis Energy Options (MEO) campaign began with similarly moderate to poor conditions. MEO attempted to remunicipalise electricity services when the concession to the private utility was due to be renewed as a means to open the field of energy to citizens. MEO organized a coalition of social movement organizations. In this particular case, the city council would need to vote to put a question to authorize looking into municipalization on the ballot. The campaign focused on informing residents that there were other options in energy provision, and using various tactics to put pressure on the council, including: petitioning and educational presentations; going through the party endorsement process for the ballot initiative; choosing to run their campaign during a municipal election year; having candidates take positions on it. MEO managed to place the issue front and centre in campaigning and election discussions.

Similar to Berlin, the campaign was able to shift council opinion and built enough public and media pressure that the council had to act to adopt a new approach with the utilities. The council did not agree to put the initiative to authorize municipalization on the ballot, though. After a period of study, the city went forward with an alternative proposal for a partnership that was restricted to the two utilities and the city, called the Clean Energy Partnership (CEP), with a formal board, work plans towards meeting the city’s climate and energy goals, and a community advisory council. MEO had also built up pressure on the utilities and awareness of energy issues within the city so that the utilities agreed to some important concessions in the process of negotiating with the city, including a drastic shortening and reworking of the contract to make it possible to cancel after five years.

Here the city adopted the campaign’s renewable goals and to a certain extent its framings (if not action) around social, democratic and local elements but restricted participation in its application. It could not picture public ownership for two reasons. First, the electric utility Xcel had mobilized significant opposition, particularly from the business community. What is more, a state law in Minnesota requires that, in municipalization proceedings, cities consider lost profits to the utility, which created significant financial and legal doubt about the feasibility of going the municipalization route under those circumstances. Nevertheless, the campaign had forced the city to become involved in energy decision making, which it was not previously. Additionally, the advisory board in the CEP offered a stage for intervening in this discussion, including the pushing of several policy proposals.

The CEP does have a clear structure, moderate transparency, and supposedly clear goals, but has had mixed to scant results overall. One primary factor limiting the CEP was argumentation from the utilities that they couldn’t provide funding for partnership activities because a regulatory “non-discrimination” clause kept them from spending money preferentially in one part of their service area. Another was that a structure attempting coordination between three large bureaucratic institutions created even more institutional slow-down points. Additionally the parity of votes on the board means that things that are controversial will likely not be advanced in this space. Consequently most of the results of the CEP have been actions taken by the city, often on an original idea proposed by the advisory council. Finding a funding stream for city energy activities falls under this category, as well as the city championing a policy that would allow low-income people and renters to pay for energy upgrades out of the savings they create, on their bills (inclusive financing/PAYS^®^ model). The utilities, with city support, have made progress especially in expanding uptake of and practices of engagement around their state-mandated efficiency programs, or in sharing data with the city, but many of the larger goals have not been met.

### Jena, Germany

The Jena case began in very positive starting conditions, from a clear understanding on the need to increase participation to a generally favorable ground for such discussions in the field. Jena is one of the first cities in Germany in which a citizens’ energy cooperative acquired shares in the municipal utility company. Three local key players in the environmental policy field, with support from the local Green Party, initiated the idea of setting up a cooperative. These three actors were the current head of the finance department and former chairman of the Green Party in the city council, the former director of the municipal energy company, and the current Green deputy mayor for the environment and urban planning. A campaign of communication and fund raising took place over the next four years to finance this project. This initiative culminated in 2011, when citizens founded the Jena Citizens’ Energy Cooperative (BürgerEnergie Jena eG).

This structure, based on co-ownership, offers clear decision rules, transparent processes of decision-making and is fairly open in terms of participation as it is supposed to give direct access to citizens through the cooperative (Blanchet and Herzberg 2019). The official aim of the cooperative is to promote a decentralised, affordable, environmentally and climate-friendly energy supply based on 100% renewable energy sources. Supported by the political majority (Christian Democrats, Social Democrats and the Green Party) in the city council, the cooperative acquired a 2% share in the municipal energy company in February 2012. Through its organization and mode of operation, the cooperative makes it possible to instill principles of participatory democracy into the local energy system, such as the participation of a representative of the citizens’ cooperative in the shareholders’ assembly, or the creation of venues for debate between citizens and experts on local energy policies. The members of the cooperative bring the actors of local energy policies in direct contact with the inhabitants of Jena, who can thus discuss the functioning and strategies of the municipal company in an open manner and take note of important and sometimes even unpublished information (Herzberg and Blanchet 2020). Citizens’ meetings and general assemblies represent, according to some local political leaders, ‘a real democratic experience’, as local elites debate directly with citizens, to whom they have to justify their actions and decisions.

However, the outcome of this particular collaborative arrangement is very nuanced. The founding members of the cooperative aimed to invest systematically in renewable energy projects and to make an important contribution to the local energy transition. But the cooperative undermined its own legitimacy. At the first general meeting, a decision was made on what to do with the 4.1% interest that was to be returned to the members of the cooperative. A clear majority voted for a complete reversal of profits to the members of the cooperative at the expense of investments in renewable energy, thereby creating a substantial boundary between cooperative members and other citizens. Trapped in the democratic principle of the cooperative’s operation, the cooperative’s management faced harsh criticisms by the different local political parties (Blanchet and Herzberg 2019). In September 2015, the members of the cooperative offered the purchase of new shares in order to reach 5% of the capital of the utility. The parties of the majority coalition opposed the request on financial grounds. In 2016, the city council refused a new request, this time to sell 3% of the shares in the municipal company, arguing that the municipal company should serve the interests of citizens and not those of investors.

### Riding Sunbeams, UK

The UK case began with moderate conditions. It involved an umbrella organization for local community energy groups (Community Energy South), an experienced environmental campaigning organization (We are Possible), a leading environmental consultancy (Ricardo), two universities (University of Birmingham and University of Bristol), and Great Britain’s railway infrastructure provider (Network Rail). The group that coalesced around the creation of a community and commuter co-owned distributed solution for railway traction supply had no prehistory of cooperation (Murray and Bottrell 2017; Murray and Pendered 2019). Despite some incentives and sense of interdependence, the imbalance in terms of power was strong, especially due to the reliance on Rail Network to create a route to market for this supply solution through procurement (Nolden 2020).

In 2019, Riding Sunbeams was set up by We are Possible and Community Energy South as a company limited by guarantee (not-for-profit) to create such a route to market for community and commuter (co-)owned RES as a means to power trains. Following the termination of subsidies in 2020, the diffusion of such sustainable energy supply innovations hinges on the direct procurement of electricity, often using Power Purchase Agreements (Nolden et al. 2020). These contractual agreements necessitate collaborative governance which increase in complexity the more actors are involved.

This particular case offers a very nuanced view on institutional design as the rules of participation among the core members were clear and aimed to open the fields of energy and public transportation to citizen-based, locally produced renewables, but the whole project stumbled upon the expectations and organizational rules of Network Rail as central gatekeeper to a full roll out. The UK Department for Transport (DfT) provided several rounds of Small Business Research Initiative (SBRI) First of a Kind (FOAK) funding to support demonstration and commercialization of this innovation (Murray and Pendered 2019).

Collaborative governance underpinning the demonstration helped address several technological issues and enabled Network Rail to take solar power traction through compliance. The creation of a route to market, however, still hinges the demonstration of technical, commercial and legal feasibility at scale. Any benefit from this new form of electricity provision needs to fulfil procurement specifications of one central actor, Network Rail, in its attempt to obtain economies of scale and reduce the number of contractual obligations. This implies that the collaborative governance which was essential to develop this innovation and increase its technology readiness level (TRL) will need to be replaced by a traction power supply contract between one supplying party and Network Rail to create such a route to market. Network Rail’s prioritization of economic efficiency principles, such as economies of scale, suggests that current contractual arrangements stand in opposition to the maintenance of collaborative governance arrangements, not to mention energy democratizing ambitions championed by Riding Sunbeams’ parent companies We are Possible and Community Energy South. Collaborative governance therefore appears to play a key role in the emergence of such potentially energy democratizing socio-technical innovations but both ED elements are in danger of being side-lined when the route to market hinges upon procurement by organizations bound by principles of economic efficiency.

### Kalmar, Sweden

In comparison to countries such as Germany and Denmark, where community energy projects are much more prevalent (Ruggiero et al. 2021b), Sweden does not provide a policy framework that is conducive to these kinds of projects. Against this backdrop, it is not surprising that some community energy projects in Sweden do not have the traditional setup of grassroot initiatives but instead are a product of different (state) actor collaboration with local inhabitants (Ruggiero et al. 2021a). One example for this phenomenon is the Kalmarsund Sol community energy association. The project was developed by Kalmar Energy, the local public energy company in the Kalmar municipality in the South-East of Sweden. The municipality holds a majority of shares of Kalmar Energy, making this project effectively a case of collaborative governance between a municipality, a publicly-owned energy provider, inhabitants and local businesses.

Kalmar Energi has a long-standing history of initiating and managing community energy projects in the region, which is blessed with ample potential for wind and solar energy production. In 2006, the company started the Kalmarsund Vind project, which was de-facto a community energy project for the company’s local customers. A decade later, in 2016, the Nöbble Solar Park started operating. The Nöbble Solar Park is one of two collectively owned solar park with a capacity of 600.000 kWh/a. The following year saw the addition of the Törneby Solar Park, which added some 2.300.000 kWh/a, making it Sweden’s biggest solar park at that time.

After facilitating the construction and putting the two solar parks into operation, Kalmar Energi legally handed over the facilities to the consortium of shareholders. The consortium consists of (1) the Kalmarsund Sol association, which unites all the participating households; (2) a number of local businesses and (3) the county administration who has bought shares in the parks to power public buildings such as the local courthouse. Kalmar Energi now serves as a service provider who manages the day-to-day operation of the two solar parks. Each owner - whether they are a private household, a local business or a public body - has a limit of shares in the project they can purchase. The limit is set to 80% of the consumption of the previous year. A set of investment rules makes sure that no owner can become a majority shareholder, which in turn ensures a democratic distribution of the shares. In addition, all shareholders have one (and only one) vote when strategic decisions on the two parks are taken.

The project brought about a number of benefits to the parties involved. The municipality of Kalmar supported the project as it strengthened its green image, which can be considered a form of green city branding (Busch and Anderberg 2015). Kalmar Energi also used the project for green branding and to create stronger bonds to its customers. In addition, Kalmar Energi uses its knowledge from the establishment of the two solar parks by selling the concept to other local energy providers in Sweden. For local businesses and involved households, the project was an easy way to become prosumers and thus take an active part in the governance of the energy system.

## Discussion

Our results point to important new nuances regarding the structuring effects of the collaborative processes on existing power relations and context. By framing ED within the CG literature, we will now discuss the importance of a broader analysis of tensions as the expression of dichotomous logics within collaboratives engaged in ED processes.

From the five case studies, it is striking that the only successful project, the Kalmar case in Sweden, involves an initiative by a traditional public sector utility that moved from there towards a more open and democratic community approach that delivered on ED objectives. The other four involve bottom up initiatives challenging an incumbent system, albeit with varying degrees of conflict. The two case studies on civic participation (Berlin and Minneapolis) were co-opted by government and private institutions, ultimately leading to collaborative regimes, yet watered down to moderate or small achievements. The cases from the UK report on collaboratives that emphasized commercialization at the expense of broader democratic, social and ecological principles. We now turn to the two main dichotomous processes at play in these cases: (1) restricting versus (2) opening participation.

### Opening Participation

As two cases in agenda-setting (along with grassroots policy leadership in their later more collaborative phases), both the Berlin and Minneapolis examples were characterized by social movement coalitions demanding public ownership and democratic and local control of energy systems, especially electricity. In both cities, a window of opportunity had opened to do so as municipal contracts with commercial utilities were about to be renewed. Campaign initiators at the time perceived commercial utilities as incumbents antithetical to the renewable transition, especially a local or regional energy transition based on democratized and decentralized generation. The social movements subsequently sought to provide alternative visions of how the energy sector should be organized, based around social justice and local democracy and benefit. Their strategies towards opening relied vastly on institutional resources for civic participation, such as elections and referendum, as well as on campaigning and raising awareness for political actions. In terms of proposal, the two cases foresaw tools and organizational designs that make it possible for citizens to hold leadership accountable and to determine strategies for energy policy in their community.

The next three cases emerged in contexts that reveal similar forces at play over the question to open control over the system. The Jena case is one in which governance actually changed towards more ED. Contrary to the Berlin and Minneapolis cases, this idea evolved from planning towards implementation without much conflict yet needed four years of campaigning and raising funds among residents to empower them financially and become a formal participant in an otherwise centralized system. Similarly, in the UK, support for local and democratic control of energy supply ceased with the termination of government underwritten feed-in tariffs in 2020. Organisations seeking to promote ED through RES generation now need to sell power through power purchase agreements, which hinge upon successful CG. In the case of Riding Sunbeams the institutional actor Network Rail, Great Britain’s railway infrastructure provider, determines the contractual terms and these are unfavorable towards energy democratization.

Kalmar offers a contrast illustration of a public company choosing to open its decision-making process and benefit distribution, at least within a specific project. Eventually, the utility handed over the running and benefit of the association to local residents and business, while retaining a technical assistance and facilitative role. The municipality holds a majority of shares of Kalmar Energy, making this project effectively a case of collaborative governance between a municipality, a publicly-owned energy provider, inhabitants and local businesses.

### Restricting Participation

The cases demonstrate that democratizing principles are sometimes established on paper but often not realized in practice over the course of the collaborative process. We note that in these cases, specific participants used leadership and design as resources to restrict the scope and/or gain more control over specific processes in favour of their own interests. The failure to create a balance between collective and participants’ goals proved to be crucial.

The Minneapolis Energy Options (MEO) campaign attempted to remunicipalize electricity services when the concession was due to be renewed. MEO organized a coalition of social movements and organizations. Rather than providing a specific agenda to democratize the internal operations of a utility as in the Berlin case study, the MEO campaign raised these topics more generally in an effort to force a public conversation. The campaign was able to shift council opinion and build enough public and media pressure, which the council co-opt to propose a new approach with the commercial utilities. This approach, however, was limited against the backdrop of the campaign’s goals. The partnership that emerged was then undermined by weak funding and the board’s rules for voting and decision making.

In Berlin, dominant governmental actors used institutional rules to co-opt and shut down the movement. Most of the internal organization was democratic and inclusive. There was a steering committee, a few prominent campaign speakers and a paid campaigner, but decisions were made collectively through voting in open plenaries. There were working groups to help develop the social, ecological, and democratic elements, and volunteer lawyers to help draft the collective proposals in legal language. Despite significant and much delayed success in remunicipalizing energy distribution, however, the initiative now maintains only a handful of activists, and many of the democratic and social goals of the initiative were not instituted.

In Jena, the cooperative’s inclusive and democratic mode of functioning made it possible to instil principles of participatory democracy into the local energy system. And yet, although the founding members of the cooperative had aimed to invest systematically in renewable energy projects, the majority of its members decided to vote for a complete return of profits to the members of the cooperative instead.

Public funding enabled Riding Sunbeam and the demonstration system helped address several technological issues and enabled Network Rail to take solar power traction through compliance. But despite the capacity of the collaborative partnership to organize demonstration and proving commercial and legal feasibility, (Nolden et al. 2020) satisfying Network Rail’s principles of procurement is forcing Riding Sunbeams to abandon its aim to connect community and commuter (co-)owned RES into the traction supply network. Eventually, the project restructured as Riding Sunbeams Apollo, a company limited by shares, to attract commercial investors and create economies of scale but it is still unclear whether the abandonment ED principles is sufficient for the creation of a route to market.

In the Kalmar case, participation remained open over time. Kalmar Energi legally handed over the facilities to the consortium of shareholders. Kalmar Energi now acts as a service provider who manages the day-to-day operation of the two solar parks in the name of the collaborative. No owner can become a majority shareholder. In addition, all shareholders have one (and only one) vote when strategic decisions on the two parks are taken.

## Conclusion

While the cases offer substantial variations in terms of context and objectives, their differences pose limits to their generalisation. More cases are necessary to substantiate the potentially conflicting co-existence of opening and restricting strategies as two central dichotomous processes at play. Nevertheless, these observations highlight the challenges that ED initiatives are confronted with compared to other fields and instances of collaborative governance, where success in democratization and participation is perhaps more common. These observations also highlight the degree to which attention must be paid to the overall goals of the energy system, and the diversity of actors involved. Examining ED within CG frames highlights that ED processes are marked by fundamentally different approaches to policy, driven by opposing goals of different actors – just having a collaborative process won’t resolve those differences.

This finding might also hold for other policy areas and approaches, and shows a challenge to CG frameworks that merits further investigation. Most frameworks posit a rather linear trajectory from turbulent start to stability in the collaborative. Our observations indicate how turbulent the early phase can be, and how much attention needs to be paid to pull forces towards openness on the one hand, and restriction of participation on the other. Ultimately, all these initiatives yielded some form of CG or another, but only one of them delivered on both energy and democracy goals.

ED therefore seems to require a significantly level of attention to issues of participative facilitation, consensus building, commitment, and shared achievements. Hence, it seems advisable for public authorities to mandate CG as an outcome rather than an input. For example instead of requiring CG as a condition for successful bids to energy tenders, it should be specified as a contractual obligation. Alternatively, different goals would need to be set institutionally, legislatively, or administratively to ensure continuity in later phases of implementation.

Our investigation of ED processes highlights the need for strong network intermediation capacities, either in the hand of a central actor with a preference for ED, as in the Kalmar case, or through stronger campaigning and movement work to shift political realities (Fairchild and Weinrub 2017; Nolden et al. 2020). On a similar note, these findings corroborate the point made by Emerson and Gerlak (2014) on the crucial need for adaptive capacity to sustain collaborative arrangements. Against the backdrop of their argument, our observations emphasize the need for multi-level leadership. Our analysis also underlines the need to not only invest in advocacy, but also in supporting and mobilizing stakeholders for change as their interests evolve amidst collaborative change. Only then can we hope to effectively and sustainably address conflicts in collaborative governance processes, which would become more prevalent in a low-carbon energy transition.
